# A Naked-Eye Visual Reverse Transcription Loop-Mediated Isothermal Amplification with Sharp Color Changes for Potential Pen-Side Test of Foot-and-Mouth Disease Virus

**DOI:** 10.3390/v14091982

**Published:** 2022-09-07

**Authors:** Jie Zhang, Qian Hou, Weimin Ma, Danian Chen, Weibing Zhang, Ashenafi Kiros Wubshet, Yaozhong Ding, Miaomiao Li, Qian Li, Jiao Chen, Junfei Dai, Guohua Wu, Ziteng Zhang, Alexei D. Zaberezhny, Zygmunt Pejsak, Kazimierz Tarasiuk, Muhammad Umar Zafar Khan, Yang Wang, Jijun He, Yongsheng Liu

**Affiliations:** 1 Hebei Key Laboratory of Preventive Veterinary Medicine, College of Animal Science and Technology, Hebei Normal University of Science & Technology, Qinhuangdao 066004, China; 2 State Key Laboratory of Veterinary Etiological Biology, Lanzhou Veterinary Research Institute, Chinese Academy of Agricultural Sciences, Lanzhou 730046, China; 3 Federal State Budgetary Institution, All-Russian Research and Technological Institute of Biological Industry (VNITIBP), Moscow 141142, Russia; 4 Department of Infectious and Parasitic Diseases, University Center of Veterinary Medicine Jagiellonian University—Agriculture Universities, 31-120 Krakow, Poland; 5 Institute of Microbiology, University of Agriculture Faisalabad, Punjab 38040, Pakistan

**Keywords:** foot-and-mouth disease virus, loop-mediated isothermal amplification, naked-eye visualization, pen-side test, 3D gene

## Abstract

Visual loop-mediated isothermal amplification (LAMP) is qualified to be applied in the field to detect pathogens due to its simplicity, rapidity and cost saving. However, the color changes in currently reported visual reverse transcription LAMP (RT-LAMP) for foot-and-mouth disease virus (FMDV) detection are not so obvious to the naked eye, so interpretation of results is troublesome. In this study, a new naked-eye visual RT-LAMP to detect all seven distinct serotypes of FMDV was established based on the 3D genes by using pH-sensitive neutral red as the indicator, rendering a sharp contrast of color changes between the negative (light orange) and the positive (pink). Analytical sensitivity tests showed that the detection limit of the visual RT-LAMP was 10^4^ copies/µL while those were 10^3^ and 10^4^ copies/µL for the RT-qPCR and conventional RT-PCR methods, respectively. Specificity tests proved that the established visual RT-LAMP assay had no cross-reactivity with other common livestock viruses. Furthermore, the analysis of 59 clinical samples showed 98.31% and 100% concordance with the RT-qPCR and the RT-PCR, respectively. The pan-serotypic FMD visual RT-LAMP assay could be suitable for a pen-side test of all seven serotypes of FMDV because the results could be easily distinguished by the naked eye without the requirement of complicated instruments and professional technicians. Hence, the novel method may have a promising prospect in field tests which exert an important role in monitoring, preventing, and controlling FMD, especially in regions with no PCR or qPCR instrument available.

## 1. Introduction

Foot-and-mouth disease (FMD), caused by the foot-and-mouth disease virus (FMDV), is a highly contagious infectious vesicular disease of cloven-hoofed animals including cattle, pigs, sheep, and many wildlife species. FMDV is currently divided into seven serotypes, namely O, A, C, Asia1, and SAT (Southern African Territory) 1, 2 and 3. Although serotype C FMDV is now considered to be extinct outside of the laboratory, other serotype viruses remain endemic in Africa and southern Asia [1]. In China, FMDV serotypes A and O, are responsible for the outbreaks and have brought massive losses to both the social economy and the livestock industry [2]. According to the data statistics, US$6.5–US$21 billion is spent on production losses and vaccination of FMDV every year globally [3].

FMDV is a member of the Picornaviridae family which is a group of non-enveloped, single-strand, positive-sense small RNA viruses [4]. FMDV genome is surrounded by an icosahedral capsid and is approximately 8500 nt consisting of two untranslated regions (UTR) as well as a single open reading frame (ORF). The ORF encodes 4 structural proteins, namely VP1, VP2, VP3, and VP4, which assemble into the icosahedral capsid, and 11 non-structural proteins, such as Lab, Lb, 2A, 2B, 2C, 3A, 3B1, 3B2, 3B3, 3C and 3D [5]. The 3D gene encodes the viral RNA-dependent RNA polymerase (RdRP) and possesses highly conservative features in the sequence of different serotypes and subtypes of FMDV. Therefore, it allows us to establish FMDV pan-detection methods, such as the RT-qPCR recommended by World Organization for Animal Health (WOAH), the conventional RT-PCR, loop-mediated isothermal amplification (LAMP) and recombinase polymerase amplification (RPA) while the VP1 coding gene is the target for typing and molecular epidemiology [6].

Currently, WOAH recommends conventional and quantitative RT-PCR for fast diagnosis. However, those methods are heavily dependent on specific types of equipment and professionals which make them unsuitable for pen-side detection [7]. FMDV can rapidly spread among herds, and therefore pen-side tests characterized by time-saving, simple operation, and inexpensive instrument requirements for virus detection would be helpful in epidemic control and prevention, and eventually disease eradication [8]. LAMP, a gene amplification technique relying on the strand displacement function of Bst DNA polymerase under constant temperature conditions, is largely different from traditional nucleic acid amplification methods, such as PCR and qPCR under variable temperatures [9]. LAMP can significantly shorten the time for obtaining results (less than 1 h) and greatly decrease the dependence on professional amplifiers (heat block enough). What is more, a set of six primers (outer primers F3, B3, inner primers FIP, BIP, and loop primers Floop, Bloop) specially attaches to eight different regions of the target gene to make LAMP assay highly sensitive and specific, and naked-eye visualization based on the generation of a huge amount of amplicons [10,11]. This unique superiority confers LAMP as the ideal approach for epidemic pen-side detection.

Up to now, several different kinds of reverse transcription LAMP (RT-LAMP) assays for FMDV detection have been established, such as the classical RT-LAMP with the appearance of a ladder-like pattern of bands when amplicons were electrophoresed on an agarose-gel [12,13]; RT-LAMP with the aid of a real-time turbidimeter to detect amplification products [14]; RT-LAMP performed on the portable real-time fluorometer for the field application and confirmed the results according to the anneal derivative temperature (Ta) [15]; probe-based real-time RT-LAMP assay [7] or the visual RT-LAMP (RT-vLAMP) examined with a directly naked-eye observation by adding SYBR Green I or hydroxy naphthol blue (HNB) as a colorimetric indicator [16,17]. Among those assays, the visual RT-LAMP is in the spotlight due to its potential for field in-site tests. However, the lack of sharp color changes makes them difficult to be distinguished with the naked eye, limiting their pen-side test application.

In this research, we established a new visual RT-LAMP assay based on the conserved 3D gene to achieve rapid and convenient detection of all seven serotypes (pan-serotypic) of FMDV at a pen-side level by providing a sharp color shift. The pre-added chromogenic agent-neutral red could visualize the detection results by presenting a striking color contrast between positive and negative reactions due to the pH drop based on the production of a huge amount of amplicons. The color of the positive tubes changed from light orange to pink while the negative tubes remained the same. In addition, the specificity, sensitivity, and clinical evaluation with field samples of the new visual RT-LAMP assay were investigated to confirm whether it offers reliable detection of FMDV.

## 2. Materials and Methods

### 2.1. Preparation of FMDV 3D RNA Transcript Standard

A 500 nt segment of the 3′ end region of the 3D gene with high homology between all serotypes was selected as the target region after the alignment (MEGA 7.0) [18] of the FMDV 3D sequences. A total of 39 such DNA fragments (500 bp) representing seven serotypes were commercially synthesized to establish the pan-serotypic FMDV naked-eye visual RT-LAMP. Their accession number in Genbank were shown in Table 1. The T7 sequence 5′-TAATACGACTCACTATAGGG-3′ was added to the 5′ terminal of the segment, which was used for the in vitro transcription of RNA subsequently. The 39 synthesized 3D gene DNA fragments were cloned into a pUC57 cloning vector (Tsingke Biotechnology Co., Ltd., Xian, China). The 3D-pUC57 recombinant plasmids were extracted as the templates and then those 3D DNA fragments with T7 sequence were amplified by PCR with universal forward primers (pUC57-3DF) 5′-TAATACGACT CACTATAGGGGCATC-3′ and reverse primer (pUC57-3DR) 5′-TGCGTCACCGCACACGGCGTT-3′ under the following conditions: 95 °C, 5 min; (95 °C, 30 s; 56 °C, 30 s; 72 °C, 45 s) × 36 cycles and final extension at 72 °C for 10 min. After that, the 520 bp 3D amplicons (including the 20 bp T7 sequence) were purified using Takara MiniBest Agarose Gel DNA Extraction kit ver4.0 (Takara, Dalian, China) and then were subject to in vitro transcription. The generated RNA was cleaned according to the instruction manual (Takara, In Vitro Transcription T7 kit, Dalian, China; Takara, RNA mini cleaning kit, Dalian, China) after removing amplicon debris by enzyme digestion.

To prepare the 3D RNA transcript standard, the concentrations of the acquired RNA transcripts were determined using NanoDrop 2000 (Thermo Fisher Scientific, Wilmington, DE, USA) and the copy numbers of the RNA transcripts were calculated. Ten-fold dilutions were carried out from 10^7^ to 10^1^ copies/µL and the diluted RNA transcripts were then used as standards for determining the analytical sensitivity of the assays.

### 2.2. Viruses and Nucleic Acid Samples

The genome of the contagious pustular dermatitis virus (ORFV), sheep pox virus/goat pox virus (SPPV/GTPV), bovine viral diarrhea virus (BVDV), blue tongue virus (BTV), and classical swine fever virus (CSFV) were obtained from the infected cell culture supernatant using MiniBest Viral RNA/DNA Extraction Kit Ver.5.0 (Takara, Code No.9766, Dalian, China). The genome of the Seneca Valley virus (SVV), highly pathogenic porcine reproductive and respiratory syndrome virus (HP-PRRSV), porcine circovirus type 2 (PCV2), porcine parvovirus (PPV), pseudorabies virus (PRV), inactivated FMDVs including serotype O, A, Asia1 and SAT2, and the 36 FMDV negative clinical samples were all preserved and provided by National/WOAH FMD Reference Laboratory, Lanzhou Veterinary Research Institute, Chinese Academy of Agricultural Sciences (LVRI, CAAS). The RNAs of 21 FMDV (unknown serotype) positive clinical samples were provided by the National/WOAH FMD Reference Laboratory. The detailed information on these materials was listed in Table S1.

### 2.3. Primer Design

To ensure visual RT-LAMP assay established here was suitable for all serotypes of FMDV detection, all full-length 3D genes retrieved from Genbank were aligned by MEGA7.0 [18] and 500 nt segment at the 3′ end of 3D was selected as the primer design region. Six sets of primers were designed using Primer Explorer 5.0 (PrimerExplorer V5 Software. http://primerexplorer.jp/lampv5e/index.html, accessed on 10 June 2022) as candidate primers finally. Each set consisted of three primer pairs F3/B3:FIP/BIP:Floop/Bloop. All these primers were commercially synthesized (Tsingke Biotechnology Co., Ltd., Xian, China).

### 2.4. RT-qPCR and Conventional RT-PCR Methods

The RT-qPCR (targeting the 3D gene) assay recommended by (WOAH) (CHAPTER 3.1.8. FOOT AND MOUTH DISEASE (INFECTION WITH FOOT AND MOUTH DISEASE VIRUS). Available online: https://www.woah.org/fileadmin/Home/eng/Health_standards/tahm/3.01.08_FMD.pdf (accessed on 19 August 2022) was carried out as per the procedures using One Step (TaqMan probes) RT-qPCR Kit (MyLab Biotechnology Co., Ltd., Beijing, China). A total of 25 µL reaction system including 2× One Step RT-qPCR mix 12.5 µL, Enzyme mix 0.5 µL, 0.5 µL F primer (10 µM), 0.5 µL R primer (10 µM), 0.5 µL FAM probe (10 µM), 1 µL template RNA and 9.5 µL DNase/RNase free ddH2O was incubated in CFX96 Touch real-time PCR detection system (Bio-Rad, Foster city, CA, USA) under the following condition: 50 °C, 30 min (reverse transcription); 95 °C, 3 min; (95 °C, 10 s; 55 °C, 30 s) × 50 cycles. The results were judged by the threshold cycle (Ct) of the fluorescent reporter. Ct values below the cut-off value (CT < 40) were considered positive.

The primers with the production of 208 bp amplicon for the agarose gel-based RT-PCR targeting the 5′ end region of the 3D gene [19] were used in our study. Seven full-length 3D genes (GenBank accession number KC440882, MF461724, AF274010, MF782478, AY593845, AF540910, KF647850, representing serotype O, A, C, Asia1, SAT1, SAT2 and SAT3, respectively) were synthesized and then copied into RNA via transcription described in Section 2.1 above to serve as the template. A total of 1 µL the RNA transcript was added to the total reaction system (25 µL) consisting of 2× One Step RT-qPCR mix 12.5 µL, Enzyme mix 0.5 µL, 1 µL (10 µM) each of forward and reverse primer and 9 µL DNase/RNase free ddH_2_O. Amplification occurred under the following condition: 50 °C, 30 min (reverse transcription); 95 °C, 5 min; (95 °C, 10 s; 56 °C, 30 s; 72 °C, 45 s) × 36 cycles and a final extension at 72 °C for 10 min. The RT-PCR amplification products were detected by 2.5% agarose gel electrophoresis.

### 2.5. The Identification of ORFV, SPPV/GTPV, PRV, PCV2, BVDV, SVV, CSFV, BTV, PRRSV, and PPV by PCR or RT-PCR

For the confirmation of DNA viruses including ORFV, SPPV/GTPV, PRV, PCV2, and PPV, the PCR was conducted, and the reaction mixtures (20 µL) were comprised of Taq DNA polymerase 1 µL, 2× buffer 10 µL, F/R primer (10 µM) 1 µL, DNA template 1–2 µL and ddH_2_O up to 20 µL. The reaction tubes were incubated under the conditions described in Table S2. For the self-identification of RNA viruses including BVDV, SVV, CSFV, BTV, and PRRSV, the RT-PCR was conducted and the reaction mixtures (20 µL) were comprised of Prime Script One Step Enzyme Mix 0.8 µL, 2× One Step buffer 10 µL, F/R primer (10 µM) 1 µL, RNA template 1–2 µL and ddH_2_O up to 20 µL. The reactions were performed under the conditions described in Table S3. The primer sequences were listed in Table S4.

### 2.6. Establishment and Optimization of the Visual RT-LAMP Assay

Firstly, we established the visual RT-LAMP assay with Eva green fluorescence and 39 3D RNA transcripts representing seven FMDV serotypes by running in CFX96 Touch real-time PCR detection system (Bio-Rad). A total of 20 µL reaction system consisted of 10 µL of 2× RT-LAMP buffer mixture (Mylab Medical Technology Co., Ltd., Beijing, China), 1 µL (8 U) Bst DNA polymerase (New England Biolabs Inc., Ipswich, UK), 1 µL 20 × Eva green (Biotium Inc., Fremont, CA, USA), 0.04 µL (100 µM) each of F3 and B3 primers, 0.5 µL (100 µM) each of FIP and BIP inner primers, 0.11 µL (100 µM) each of loop primers Floop and Bloop, 1 µL 3D RNA transcript template (10^5^ copies/µL) and DNase/RNase free ddH_2_O up to 20 µL. The RT-LAMP reaction was carried out at 60 °C for 60 min. The relative fluorescence units (RFU) were proportionate to the amplification efficiency and were regarded as the selection criterion. To optimize the primers, 6 sets of candidate primers were designed based on an alignment of the 500 nt highly conserved region at the 3′ end of the 3D gene. To determine the optimal reaction temperature of the assay, temperatures from 59 °C to 66 °C at 1 °C intervals were tested. To investigate the optimal time, the reaction mixtures were incubated from 30 min to 70 min. After reaction completion, the amplicons were also subject to gel electrophoresis on 2.5% agarose gel.

### 2.7. Comparison of the Chromogenic Agents among HNB, Calcein, SYBR Green, Neutral Red, Cresol Red, and Bromothymol Blue (BTB) for the Naked-Eye Visual RT-LAMP Assay

To achieve the naked-eye visual RT-LAMP assay, HNB, calcein, SYBR green I, neutral red, cresol red, and BTB were screened to work as the indicator dye. The assay with HNB or calcein or SYBR green I was performed in a total 20 µL reaction system described previously only with the replacement of Eva green by 2 µL (1 mM) HNB or 1 µL calcein or 1 µL SYBR green I (500 µM). The naked-eye visual RT-LAMP assay with neutral red or BTB or cresol red was carried out with 2× RT-LAMP buffer mixture (pH = 8.5) (Mylab Medical Technology Co., Ltd., Beijing, China), and 1 µL (500 µM) neutral red or 1 µL BTB or 2.4 µL (500 µM) of cresol red as the dye. The other components were the same as that of the visual RT-LAMP mentioned above. All the reactions happened at 61 °C for 55 min with the primers of Set 1 as the optimal candidates and were followed by 80 °C for 5 min in the PCR Amplifier or taking out the tubes less than 20 min after the reaction completion in the isothermal heat block.

### 2.8. Analytical Sensitivity and Specificity of the Naked-Eye Visual RT-LAMP Assay

To estimate the limit of detection (LOD) of the naked-eye visual RT-LAMP assay, the 3D RNA transcript standard of each serotype of FMDV was 10-fold serially diluted in PBS, and 1 µL was used as the template to achieve a final copy number ranging from 10^7^ to 10^0^ copies, tested in triplicate, to determine LOD. The RT-PCR, and the RT-qPCR were compared by also testing the RNA standard dilutions in triplicate.

The analytical specificity for the FMDV naked-eye visual RT-LAMP was investigated by its tests of the genomic materials of ORFV, SPPV/GTPV, PRV, PCV2, BVDV, SVV, CSFV, BTV, PRRSV, and PPV which represent most common viral pathogens or cause similar clinical symptoms in livestock herds in China mainland. All genomic materials were confirmed before the performance.

### 2.9. Evaluation of the Naked-Eye Visual RT-LAMP Assay with Field Samples

A total of 59 archived and field-collected clinical samples (seen in Table S1) were used to estimate the diagnostic sensitivity and specificity of the naked-eye visual RT-LAMP assay on the fast extracted genomic materials. The viral RNA was manually purified with the magnetic bead method using FineMag Quick Viral DNA/RNA Kit and magnetic stand provided by Genfine Biotech Co., LTD., (Changzhou, China) according to the instruction. Each sample was also subject to both the conventional RT-PCR [19] and the RT-qPCR recommended by WOAH to evaluate their performance.

## 3. Results

### 3.1. Establishment and Optimization of the Pan-Serotypic FMDV Visual RT-LAMP Assay

**Figure 1:**
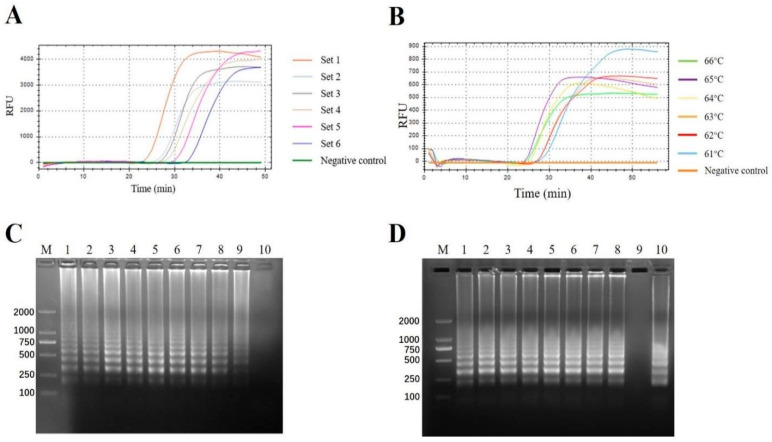
The selection of the optimal reaction conditions of the visual RT-LAMP assay. (**A**) The selection of the visual RT-LAMP primer sets measured by the fluorescent curves. Totally 6 sets of candidate LAMP primers were tested. Different primer sets were represented in different colors. (**B**) The selection of the optimal incubation temperatures measured by the fluorescent curves. The fluorescence signals under different temperatures were represented in different colors. (**C**) The selection of the optimal incubation temperatures also measured by electrophoresis. Lane M, DL2000 DNA marker (Takara, Dalian, China); Lane 1–8, different incubation temperatures subjected to the visual RT-LAMP assay (1, 59 °C; 2, 60 °C; 3, 61 °C; 4, 62 °C; 5, 63 °C; 6, 64 °C; 7, 65 °C; 8, 66 °C); Lane 9, positive control; Lane 10, negative control. (**D**) The selection of the optimal incubation time was measured by electrophoresis. The electrophoretic results of the visual RT-LAMP products were generated by incubation for a different time at 61 °C. Lane M, DL2000 DNA marker (Takara, Dalian, China); Lane 1–8, different incubation time subjected to the visual RT-LAMP assay (1, 30 min; 2, 40 min; 3, 45 min; 4, 50 min; 5, 55 min; 6, 60 min; 7, 65 min; 8, 70 min); Lane 9, negative control; Lane 10, positive control.

**Figure 2:**
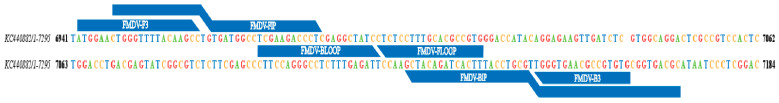
Target region of the visual RT-LAMP primers for pan-serotypic FMDV detection is shown, and reference sequence is KC440882 for FMDV type A isolate EGY 1/2012.

The 500 bp segment of the 3′ end of the 3D gene was the optimal target region and therefore, a total of 39 such DNA fragments representing seven serotypes were synthesized to establish the pan-serotypic FMDV visual RT-LAMP. Firstly, the fluorescent visual RT-LAMP with Eva green as the dye was set up to determine the reaction system by analyzing RFU. To select the ideal primers, we designed and synthesized 6 sets of candidate LAMP primers based on the 500 nt highly conserved region at the 3′ terminal of the gene. The 3D RNA transcripts (10^5^ copies/µL) (Table S1) were used as templates to screen the optimal primer set. Finally, the primers of Set 1 showed the highest amplification efficiency (Figure 1A), and the primer sequences and positions were shown in Table 2 and Figure 2. To determine the optimal reaction temperature of the assay, different reaction temperatures from 59 °C to 66 °C at 1 °C intervals were tested. The reaction mixtures were incubated at 60 °C for 60 min. The results were shown in Figure 1B,C. Combined with electrophoresis results and fluorescence amplification curve, 61 °C was selected as the best reaction temperature.

To investigate the optimal time of the visual method, the reaction mixtures were incubated from 30 min to 70 min. The electrophoresis results (Figure 1D) indicated that the brightest band occurred after 55 min. Thus, we chose 55 min as the reaction time of this assay.

### 3.2. The Selection of Chromogenic Agents for the Pan-Serotypic FMDV Naked-Eye Visual RT-LAMP Assay

**Figure 3:**
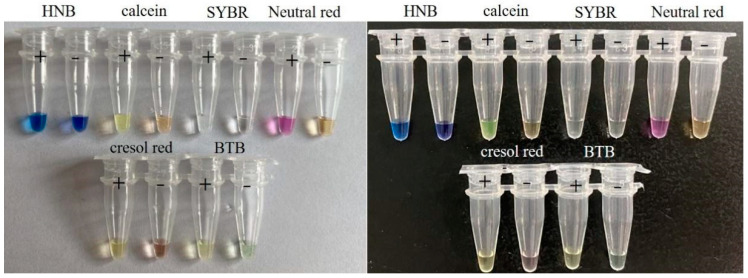
The selection of the indicator dye for the naked-eye visual RT-LAMP assay. The comparison of the color change between the positive and negative reactions of the assay with six different indicator dyes including HNB, calcein, SYBR green I, neutral red, cresol red, and BTB. The results were presented in both white and black backgrounds with positive and negative reactions. Positive reactions were shown by “+” and negative reactions were shown by “−”.

For naked-eye visual detection, indicator dye plays an important role in the assay. In our study, to acquire the ideal indicator dye for the naked-eye visual RT-LAMP assay, the color rendering effect of six dyes (neutral red, cresol red, HNB, calcein, SYBR green I, and BTB) was tested and compared by detecting the 3D gene RNA transcript. As shown in Figure 3, the neutral red presented the sharpest color change running from light orange to pink. Here, pink indicated a positive reaction while the negative remained light orange. The results of cresol red (yellow to light pink), HNB (sky blue to violet), calcein (yellow-green to yellow), SYBR green I (light yellow to achromatic color), and BTB (light green to light yellow) were all more or less relative difficult to be distinguished by naked eyes because of less difference of the color change between the positive and negative reactions.

### 3.3. Analytical Specificity and Sensitivity of the Naked-Eye Visual RT-LAMP Assay

**Figure 4:**
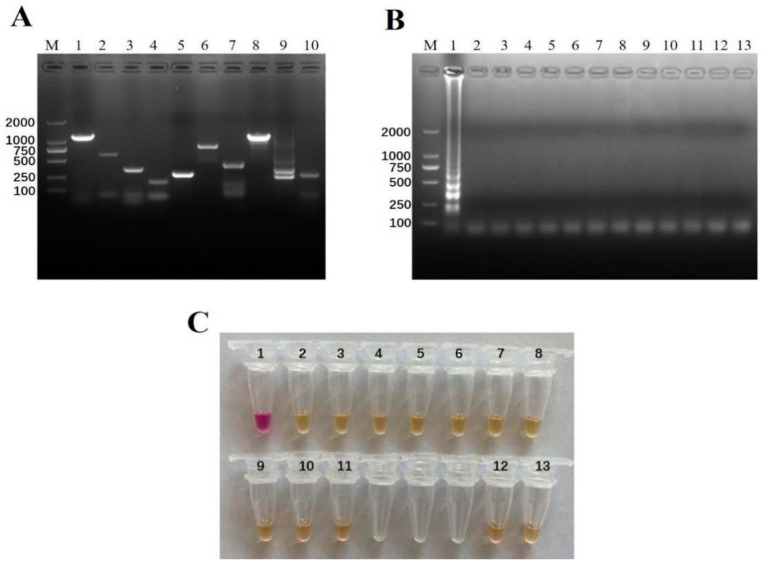
The analytical specificity of the naked-eye visual RT-LAMP assay. (**A**) Electrophoresis of self-identification of the viruses for cross-reaction analysis by the specific PCR or RT-PCR. Lane 1–10 were ORFV (1137 bp), SPPV (600 bp), PRV (356 bp), PCV2 (250 bp), BVDV (300 bp), SVV (800 bp), CSFV (424 bp), BTV (1200 bp), PRRSV (245 bp, 335 bp) and PPV (445 bp). Lane M, DL2000 DNA marker (Takara, Dalian, China). The identified viruses above were subject to the naked-eye visual RT-LAMP assay for FMDV detection. The amplified products were shown by agarose gel electrophoresis (**B**) and naked-eye examination in ambient light (**C**), respectively. Among them, No. 1–11 represented FMDV, ORFV, SPPV, PRV, PCV2, BVDV, SVV, CSFV, BTV, PRRSV, and PPV, respectively. No. 12–13 represented cell culture supernatant and ddH_2_O as the negative controls. M: DL2000 DNA marker (Takara, Dalian, China).

To confirm the specificity of the naked-eye visual RT-LAMP in detecting pan-serotypic FMDV, we detected the cross-reactivity with other nucleic acids of the viruses which can lead to similar clinical symptoms to FMD, such as ORFV, SPPV/GTPV, BVDV, and BTV. Furthermore, the genomes of the common viral pathogens of swine disease such as PRV, PCV2, PPV, CSFV, SVV, and PRRSV were also tested. Before the experiments, all the viral genomic materials were confirmed by using the specific PCR or RT-PCR followed by amplicon sequencing to prove their qualification (Figure 4A). The detection results indicated that the newly-established pan-serotypic FMDV naked-eye visual RT-LAMP assay did not have cross-reactions with BVDV, ORFV, SPPV/GTPV, SVV, BTV, PRRSV, CSFV, PRV, PPV, and PCV2, showing good analytical specificity (Figure 4B,C).

**Figure 5:**
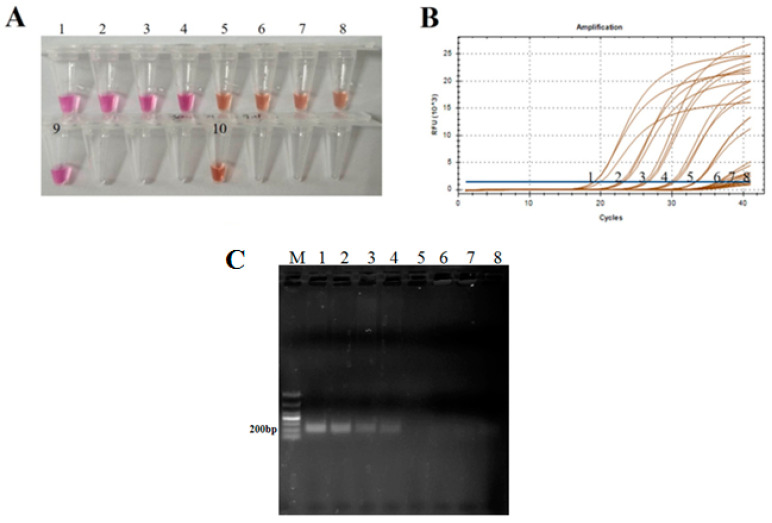
The comparison of analytical sensitivity of the visual naked-eye RT-LAMP assay between the RT-qPCR and conventional RT-PCR. LOD of the naked-eye visual RT-LAMP (**A**), the RT-qPCR (**B**), and the RT-PCR (**C**) assays for the amplification of FMDV 3D gene RNA transcript standards. Tubes, lines, and lanes 1–8 represent ten-fold serial dilutions (from 10^7^ to 10^0^ copies/µL) of the RNA transcripts. Tube 9 and 10 represent positive and negative control, respectively.

A series of ten-fold dilutions of RNA transcripts of the 3D genes of seven serotypes of FMDV were carried out and then the resulting 3D RNA transcripts were used as the templates for analytical sensitivity detection. At the same time, all the diluted 3D transcripts were subject to the conventional RT-PCR and the RT-qPCR methods. The results showed that the LOD of the naked-eye visual RT-LAMP was 10^4^ copies/µL which was the same sensitivity as the RT-PCR but 10 times lower than the LOD of the RT-qPCR (10^3^ copies/µL) (Figure 5A–C).

### 3.4. Evaluation of the Naked-Eye Visual RT-LAMP Assay with Field Samples

The clinical validation of the naked-eye visual RT-LAMP assay was performed using RNA manually isolated with the magnetic bead method from 59 clinical samples (Table S1). Among these clinical samples, 23 were positive for the RT-qPCR while 22 of the 23 RT-qPCR positive ones were also confirmed positive by both the visual RT-LAMP and the RT-PCR assay. The Ct value of sample No. 9 which failed to be tested positive by the visual RT-LAMP and the RT-PCR was 34.6. The results were shown in Table 3 and Table S5. The visual RT-LAMP established here presented 98.31% and 100% concordance with the RT-qPCR and the RT-PCR.

## 4. Discussion

At present, FMD is still one of the main influential diseases in the livestock industry in China which results in a substantial economic burden. Recently, a new strain of serotype O lineages, O/ME-SA/Ind2001, has been discovered in China in 2017, indicating the deficiencies existing in our prevention and control system, especially in border areas [20]. Furthermore, FMDV serotype SAT 1–3 is a persistent risk of being introduced into China, which is a potential threat to the safety of animal husbandry [21]. As FMD-susceptible species are easy to be infected in groups, the less costly, time-saving, and convenient pen-side diagnostic tools could lay a solid foundation for the early detection and prevention of the spread of the mutual infection of FMDV.

In our study, we established a naked-eye visual RT-LAMP assay for the detection of the seven distinct serotypes of FMDV based on the highly conserved region at the 3′ terminal of the 3D gene. Our visual RT-LAMP could be conducted at 61 °C in less than 1 h. In comparison with the variable temperature amplification methods such as PCR and qPCR, the constant amplification can be carried out in a cheap heat block or a regular water bath, conferring LAMP the potential to meet point-of-care testing (POCT). Among all sorts of LAMP methods, naked-eye visual detection outperforms others in terms of POCT in less developed regions. To realize the visualization of the detection results, we screened several different indicator dyes that almost represent all kinds of dyes including metal-sensitive dyes, intercalating dyes, and pH-sensitive dyes. As the results were shown, neutral red outperformed other dyes such as HNB, calcein, SYBR green I, cresol red, and BTB, which made this method very suitable for the pen-side test. In addition, to simplify the operation and avoid contamination, neutral red was added together with other reagents to establish a one-step reaction system.

Theoretically, RT-LAMP possesses high sensitivity due to the particularity of the primers including outer, inner, and Loop primers, and the features of Bst polymerase enzyme with high efficient strand displacement [22,23]. To prove this, a comparative evaluation of analytical and clinical sensitivity was carried out among the naked-eye visual RT-LAMP, the conventional RT-PCR referenced [19] and the RT-qPCR recommended by WOAH. Among the three methods, our visual RT-LAMP and the RT-qPCR target the 3′ end region of the 3D gene, and the RT-PCR targets the 5′ terminal of the 3D gene. A ten-fold dilution series (10^7^–10^0^ copies/µL) of the seven full-length 3D RNA transcripts of FMDV representing seven distinct serotypes and the 59 clinical specimens were subject to the three methods at the same time. LOD of our visual RT-LAMP, the RT-qPCR, and the RT-PCR were 10^4^, 10^3^ and 10^4^ copies/µL, respectively, showing a similar sensitivity between the visual RT-LAMP and the RT-PCR but around ten-fold lower than the RT-qPCR. Usually, the LOD of PCR is around 10 to 100 times lower than that of qPCR. As for clinical sample testing, our visual RT-LAMP showed the same detection capability for FMDV positive materials (22/23) as RT-PCR with 1 missing (No. 9 with Ct value of 34.6) when compared with the RT-qPCR examination. Those results indicated that the visual RT-PCR has a similar detection sensitivity as the conventional RT-PCR. The reason for the lower LOD than that of the RT-qPCR may be caused by the mismatching LAMP primers. Although the selected fragment at the 3′ end of the 3D gene is a highly conserved region, there are still some mismatches, which may lead to incomplete binding between primers and templates and result in lower LOD. Furthermore, naked-eye examination is usually less sensitive than a fluorescent signal of qPCR, which may be another main factor giving risen to lower LOD of visual LAMP.

To verify whether the established method could successfully distinguish FMDV from other viruses (ORFV, SPPV/GTPV, and SVV) causing vesicular diseases, some common swine viruses (PRRSV, CSFV, PRV, PPV, and PCV2) and some common bovine viruses (BVDV and BTV), the genomes of these viruses isolated from cytotoxic suspension were tested using the visual RT-LAMP assay. The pan-serotypic FMDV naked-eye visual RT-LAMP assay established here did not show cross-reaction with ORFV, SPPV/GTPV, SVV, PRRSV, CSFV, PRV, PPV, PCV2, BVDV, and BTV, showing good analytical specificity. Due to the extinction of swine vesicular disease and rare cases of vesicular stomatitis in China, these two pathogens were excluded from the specificity analysis in our study. The 3′ end of 3D is so well-conserved that several FMDV pan-detection methods with satisfactory specificity (see details in review article [6]) were successfully developed based on this region. In addition, there was no cross-reaction with the African swine fever virus which first appeared in the Autumn of 2018 in China [24].

The pan-serotypic FMDV naked-eye visual RT-LAMP established in this study generally showed good concordance with the RT-qPCR and the RT-PCR when they were used to test nucleic acids quickly extracted from clinical samples by magnetic bead method with on-site application potency. In comparison with the RT-PCR and the RT-qPCR, the naked-eye visual RT-LAMP is more suitable for pen-side tests because it has more distinct advantages, such as ease of use (one-step reaction system), less-professional operators (no result data processing and naked-eye examination only), cheap instruments (heat block or water bath only), and rapidity (1 h reaction period). Furthermore, we have been working to achieve more simple isolation of genomes from a clinical specimen and dry powder of the reaction mixture which are two main challenges to be solved before the pan-serotypic FMDV naked-eye visual RT-LAMP is applied in the field, especially in the undeveloped region.

## 5. Conclusions

A naked-eye visual RT-LAMP method with neutral red as the indicator dye for detecting seven serotypes of FMDV was established based on the highly conserved region at the 3′ end of the 3D genes. The visual RT-LAMP was comparable to the conventional RT-PCR [19] and the RT-qPCR recommended by WOAH in terms of their sensitivity and specificity. The convenient mode of operation, simple equipment such as heat block, and naked-eye observable test results of the visual RT-LAMP assay make it a valuable method with potential application in FMDV pen-side tests, especially in device-limited areas.

## Figures and Tables

**Table 1:** Accession number in Genbank for 39 3D genes representing seven serotypes of FMDV.

A	O	C	Asia1	SAT1	SAT2	SAT3
KT968663	MF461724	AF274010	MF782478	AY593845	AF540910	KF647850
KC588943	KY234502	AJ007572	MF372125	MF678826	AY593849	KJ820999
KC440882	KC503937	AY593805	MG372731	MN116689	JX014255	KX375417
MG923580	JN998085	AY593810	AY304994	KM268899	KJ144918	MH053341
MN116688	MN250318			AY593839	KJ676543	KF647849
KY322680	AY593823				KJ144904	MG372727
	LC456871					
	JQ973889					

**Table 2:** The primer sequences for the visual RT-LAMP for pan-serotypic FMDV detection.

Primer	Type	Position ^a^	Sequence (5′-3′) ^b^
FMDV-F3	Forward outer	6942–6963	ATGGAACTGGGTTTTACAARCC
FMDV-B3	Reverse outer	7161–7145	CACACGGCGTTCACCCA
FMDV-Floop	Forward Loop	7013–6995	CACGGCGTGCAAAGGAGAG
FMDV-Bloop	Reverse Loop	7096–7117	CTTCCAGGGCCTCTTTGAGATT
FMDV-FIP (F1c+TTTT+F2)	Forward inner	6985–6966 7027–7047	CCTGCCACGGAGATCAACTTC**TTTT**TGATGGCCTCGAAGACCCTC
FMDV-BIP (B1c+TTTT+B2)	Reverse inner	7092–7071 7144–7122	ACGAGTACCGGCGTCTCTTYGA**TTTT**ACGCAGGTAAAGTGATCTGTAGC

^a^ Position numbers are based on complete genome of FMDV type A isolate EGY 1/2012 (GenBank accession number KC440882). ^b^ Boldface indicates TTTT spacer between Fc1 and F2 or B1c and B2.

**Table 3:** Results of 59 clinical samples tested by the naked-eye visual RT-LAMP, RT-PCR, and RT-qPCR for FMDV detection.

Sample No.	RT-qPCR	The Naked-Eye Visual RT-LAMP	RT-PCR
1–8, 10–23	+	+	+
9	+	−	−
24–59	−	−	−

Notes: Positive samples were marked “+” and negative samples were marked “−”. RT-qPCR recommended by WOAH and the naked-eye visual RT-LAMP target the 3′ end of the 3D gene while RT-PCR described in [19] targets the 5′ end of the 3D gene.

## Data Availability

The datasets used and/or analyzed during the current study are obtained and available from the corresponding authors on a reasonable request.

## Supplementary Materials

The following supporting information can be downloaded at: https://www.mdpi.com/article/10.3390/v14091982/s1, Table S1: The information of the nucleic acid used in this study; Table S2: The amplification program for identification of ORFV, SPPV/GTPV, PRV, PCV3 and PPV; Table S3: The amplification program for identification of BVDV, SVV, CSFV, BTV and PRRSV; Table S4: The information of the primers used for virus self-identification; Table S5. Test results of 59 clinical samples.
