# Supplementary material for: A Naked-Eye Visual Reverse Transcription Loop-Mediated Isothermal Amplification with Sharp Color Changes for Potential Pen-Side Test of Foot-and-Mouth Disease Virus
Source: Viruses. 2022 Sep 7;14(9):1982. doi: 10.3390/v14091982 (PMC9504329; doi:10.3390/v14091982)
Supplement: Supplementary file 1 [file viruses-14-01982-s001.zip › viruses-1829546-supplementary.pdf]

Table S1. The information of the nucleic acid used in this study.

No.	Nucleic Acid Type	Virus Type	Serotype
1	Genome RNA	FMDV	Asia 1
2	Genome RNA	FMDV	SAT 2
3	Genome RNA	FMDV	Unknown
4	Genome RNA	FMDV	Unknown
5	Genome RNA	FMDV	Unknown
6	Genome RNA	FMDV	Unknown
7	Genome RNA	FMDV	Unknown
8	Genome RNA	FMDV	Unknown
9	Genome RNA	FMDV	Unknown
10	Genome RNA	FMDV	Unknown
11	Genome RNA	FMDV	Unknown
12	Genome RNA	FMDV	Unknown
13	Genome RNA	FMDV	Unknown
14	Genome RNA	FMDV	Unknown
15	Genome RNA	FMDV	Unknown
16	Genome RNA	FMDV	Unknown
17	Genome RNA	FMDV	Unknown
18	Genome RNA	FMDV	Unknown
19	Genome RNA	FMDV	Unknown
20	Genome RNA	FMDV	Unknown
21	Genome RNA	FMDV	Unknown
22	Genome RNA	FMDV	Unknown
23	Genome RNA	FMDV	Unknown
24	Genome RNA	/	/
25	Genome RNA	/	/
26	Genome RNA	/	/
27	Genome RNA	/	/
28	Genome RNA	/	/
29	Genome RNA	/	/
30	Genome RNA	/	/
31	Genome RNA	/	/
32	Genome RNA	/	/
33	Genome RNA	/	/
34	Genome RNA	/	/
35	Genome RNA	/	/
36	Genome RNA	/	/
37	Genome RNA	/	/
38	Genome RNA	/	/
39	Genome RNA	/	/
40	Genome RNA	/	/
41	Genome RNA	/	/
42	Genome RNA	/	/
43	Genome RNA	/	/
44	Genome RNA	/	/
45	Genome RNA	/	/
46	Genome RNA	/	/
47	Genome RNA	/	/
48	Genome RNA	/	/

49	Genome RNA	/	/
50	Genome RNA	/	/
51	Genome RNA	/	/
52	Genome RNA	/	/
53	Genome RNA	/	/
54	Genome RNA	/	/
55	Genome RNA	/	/
56	Genome RNA	/	/
57	Genome RNA	/	/
58	Genome RNA	/	/
59	Genome RNA	/	/
60	3D RNA Transcripts	FMDV	A
61	3D RNA Transcripts	FMDV	A
62	3D RNA Transcripts	FMDV	A
63	3D RNA Transcripts	FMDV	A
64	3D RNA Transcripts	FMDV	A
65	3D RNA Transcripts	FMDV	A
66	3D RNA Transcripts	FMDV	O
67	3D RNA Transcripts	FMDV	O
68	3D RNA Transcripts	FMDV	O
69	3D RNA Transcripts	FMDV	O
70	3D RNA Transcripts	FMDV	O
71	3D RNA Transcripts	FMDV	O
72	3D RNA Transcripts	FMDV	O
73	3D RNA Transcripts	FMDV	O
74	3D RNA Transcripts	FMDV	Asia 1
75	3D RNA Transcripts	FMDV	Asia 1
76	3D RNA Transcripts	FMDV	Asia 1
77	3D RNA Transcripts	FMDV	Asia 1
78	3D RNA Transcripts	FMDV	C
79	3D RNA Transcripts	FMDV	C
80	3D RNA Transcripts	FMDV	C
81	3D RNA Transcripts	FMDV	C
82	3D RNA Transcripts	FMDV	SAT 1
83	3D RNA Transcripts	FMDV	SAT 1
84	3D RNA Transcripts	FMDV	SAT 1
85	3D RNA Transcripts	FMDV	SAT 1
86	3D RNA Transcripts	FMDV	SAT 1
87	3D RNA Transcripts	FMDV	SAT 2
88	3D RNA Transcripts	FMDV	SAT 2
89	3D RNA Transcripts	FMDV	SAT 2
90	3D RNA Transcripts	FMDV	SAT 2
91	3D RNA Transcripts	FMDV	SAT 2
92	3D RNA Transcripts	FMDV	SAT 2
93	3D RNA Transcripts	FMDV	SAT 3
94	3D RNA Transcripts	FMDV	SAT 3
95	3D RNA Transcripts	FMDV	SAT 3
96	3D RNA Transcripts	FMDV	SAT 3
97	3D RNA Transcripts	FMDV	SAT 3
98	3D RNA Transcripts	FMDV	SAT 3
99	Genome DNA	ORFV	/

100	Genome DNA	SPPV	/
101	Genome RNA	BVDV	/
102	Genome RNA	BTV	/
103	Genome RNA	CSFV	/
104	Genome RNA	SVV	/
105	Genome RNA	HP-PRRSV	/
106	Genome DNA	PCV3	/
107	Genome DNA	PPV	/
108	Genome DNA	PRV	/

Table S2. The amplification program for identification of ORFV, SPPV/GTPV, PRV, PCV3 and PPV.

Steps	ORFV	SPPV/GTPV	PRV	PCV3	PPV
1			95°C, 5min		
2			95°C, 30s		
3	55°C, 30s	54°C, 30s	57°C, 30s	54°C, 30s	50°C, 30s
4	72°C, 90s	72°C, 40s	72°C, 30s	72°C, 30s	72°C, 40s
5			72°C, 5min		

36 Cycles were repeated from step 2 to 4.

Table S3. The amplification program for identification of BVDV, SVV, CSFV, BTV and PRRSV.

Steps	BVDV	CSFV	SVV	BTV	PRRSV
1			50°C, 30min		
2			95°C, 5min		
3			95°C, 30s		
4	55°C, 30s	58°C, 30s	55°C, 30s	58°C, 30s	58°C, 30s
5	72°C, 30s	72°C, 20s	72°C, 50s	72°C, 90s	72°C, 90s
6			72°C, 5min		

36 Cycles were repeated from step 3 to 5.

Table S4. The information of the primers used for virus self-identification.

Primer	Sequences	Product size (bp)
ORFV-F	5′-AAAGGATCCATGTGGCCGTTCTCCTCCAT-3′	1137
ORFV-R	5′-AAACTCGAGTTAATTTATTGGCTTGCAGA-3′	
SPPV/GTPV-F	5′-GCCTCGAGATGGATGATGAT-3′	600
SPPV/GTPV-R	5′-GCGGATCCACTTTTTTCTACAGCTCT-3′	
PRV-F	5′-GCGTGTACTGCGACTGCGTGTT-3′	356
PRV-R	5′-CGACCTGGCGTTTATTAACCGAGA-3′	
PCV3-F	5′-ACGGTGGGGTCATATGTGTT-3′	250
PCV3-R	5′-CGCCTGGACCACAAACAC-3′	
PPV-F	5′-GAATAGGATGCGAGGAAAG-3′	445
PPV-R	5′-GTGGAAATCTGAGAGTCTGT-3′	
BVDV-F	5′-CTAGCCATGCCCTTAGTAGGACTA-3′	300
BVDV-R	5′-CAACTCCATGTGCCATGTACAGCA-3′	
CSFV-F	5′-ATTTGGTTCAGGGCCTCC-3′	424
CSFV-R	5′-CTCCGAACAATGGTCTCCC-3′	
SVV-F	5′-AGGCGGGTAACACTGACACCGA-3′	800
SVV-R	5′-TGTCAGAAGCAGGACCAGGATTG-3′	
BTV-F	5′-GTTAAAAATCTATAGATGG-3′	1200
BTV-R	5′-GTAAGTGTAATCTAAGAG-3′	
PRRSV-F	5′-GCTCCGCGCAGGAAGGTCA-3′	335/245
PRRSV-R	5′-GTGCGTCAGCGTTGTTGTC-3′	

Table S5. Test results of 59 clinical samples.

No.	Visual RT-LAMP	RT-qPCR	RT-PCR
1	+	+	+
2	+	+	+
3	+	+	+
4	+	+	+
5	+	+	+
6	+	+	+
7	+	+	+
8	+	+	+
9	−	+	−
10	+	+	+
11	+	+	+
12	+	+	+
13	+	+	+
14	+	+	+
15	+	+	+
16	+	+	+
17	+	+	+
18	+	+	+
19	+	+	+
20	+	+	+
21	+	+	+
22	+	+	+
23	+	+	+
24	−	−	−
25	−	−	−
26	−	−	−
27	−	−	−
28	−	−	−
29	−	−	−
30	−	−	−
31	−	−	−
32	−	−	−
33	−	−	−
34	−	−	−
35	−	−	−
36	−	−	−
37	−	−	−
38	−	−	−
39	−	−	−
40	−	−	−
41	−	−	−
42	−	−	−
43	−	−	−
44	−	−	−
45	−	−	−
46	−	−	−
47	−	−	−
48	−	−	−

49	–	–	–
50	–	–	–
51	–	–	–
52	–	–	–
53	–	–	–
54	–	–	–
55	–	–	–
56	–	–	–
57	–	–	–
58	–	–	–
59	–	–	–

Positive samples were marked "+" and negative samples were marked "–".
